# Supplementary material for: Molecular basis for the binding and selective dephosphorylation of Na+/H+ exchanger 1 by calcineurin
Source: Nat Commun. 2019 Aug 2;10:3489. doi: 10.1038/s41467-019-11391-7 (PMC6677818; doi:10.1038/s41467-019-11391-7)
Supplement: Supplementary file 1 — Supplementary Information [file 41467_2019_11391_MOESM1_ESM.pdf]

Supplementary Information for

Molecular basis for the binding and selective dephosphorylation of Na^+/H^+ exchanger 1 by calcineurin

Ruth Hendus-Altenburger[1#], Xinru Wang[2,3#], Lise M. Sjøgaard-Frich[4], Elena Pedraz-Cuesta[4], Sarah R. Sheftic[2], Anne H. Bendsøe[1,4], Rebecca Page[2*], Birthe B. Kragelund[1*], Stine F. Pedersen[4*], Wolfgang Peti[2*]

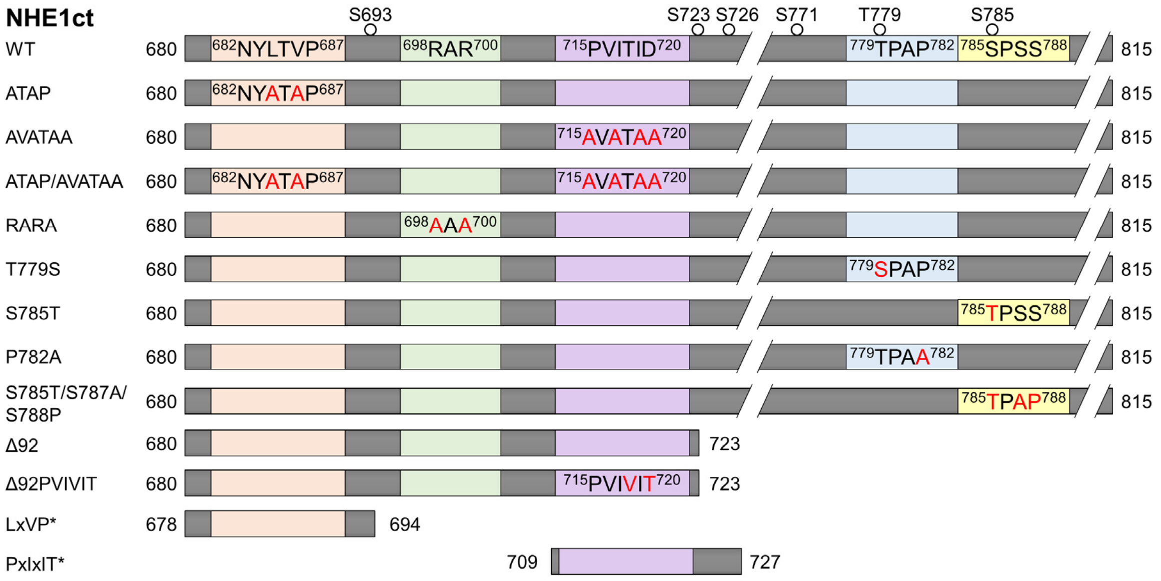

Supplementary Figure 1. *NHE1 protein variants used in this study; *peptides.*

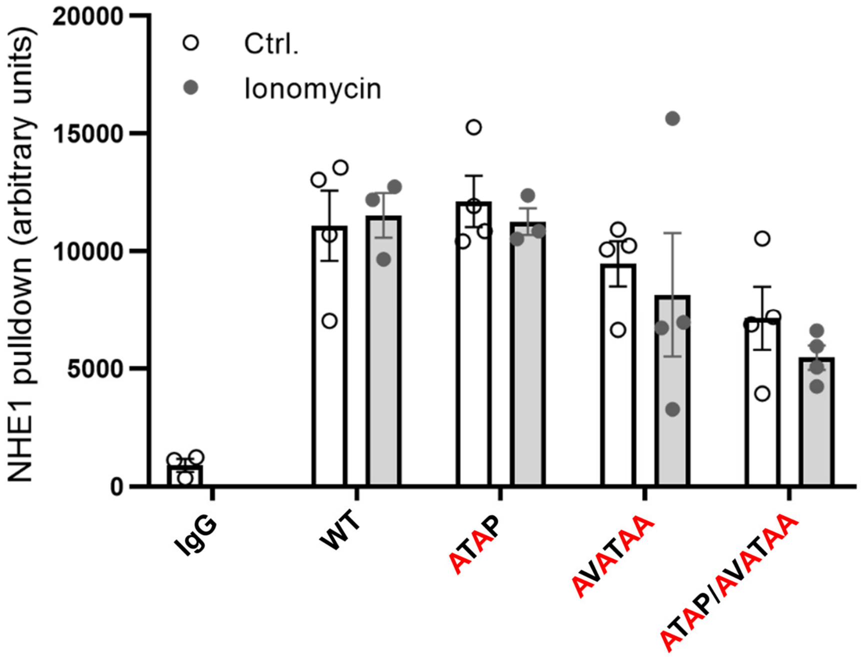

Supplementary Figure 2. *Quantification of NHE1 co-immunoprecipiation data in Figure 2H.* Data are shown as mean NHE1 band intensity, with S.E.M. error bars, of 4 independent experiments. No significant differences between WT NHE1 and variants as assessed by two-way ANOVA (*n=4*).

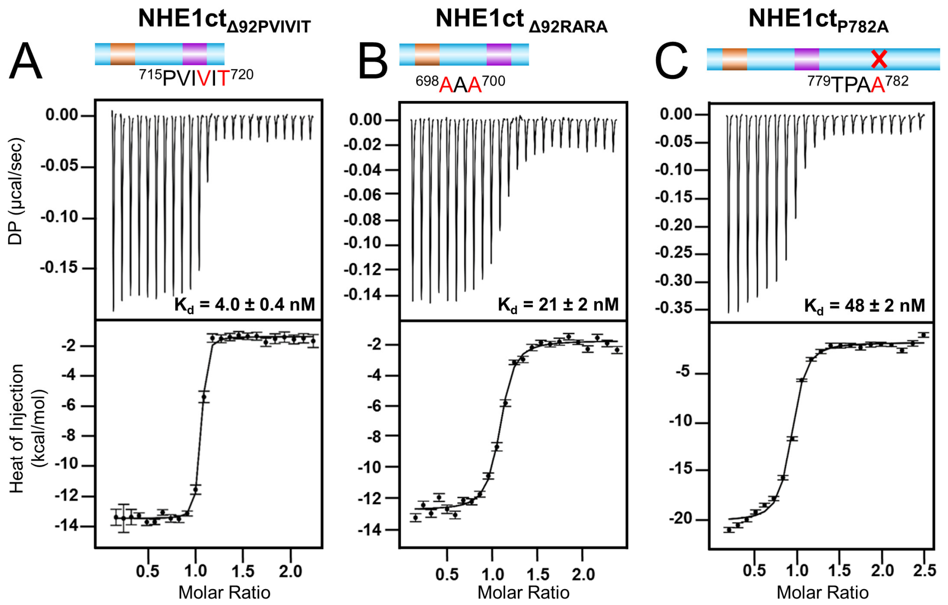

Supplementary Figure 3. *Mutating CN docking motifs alters NHE1 binding affinity for CN.*
ITC data for CN with (**A**) NHE1ct$_{\Delta92PVIVIT}$ (^{715}PVITID^{720} to PVIVIT) (**B**) NHE1ct $_{\Delta92RARA}$ (^{698}RAR^{700} to AAA) and (**C**) NHE1ct$_{P782A}$. Reported values are the average and standard deviation for replicated measurements (*n=3, 2 and 2*, respectively for A, B and C). Source data are provided as a Source Data file.

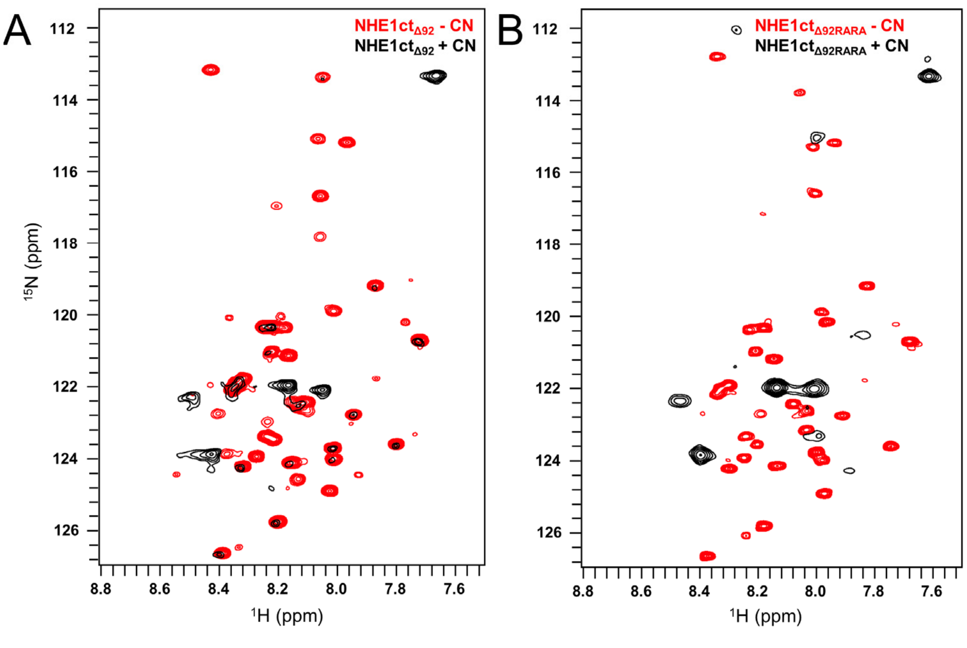

Supplementary Figure 4. *Mutating R698 and R700 in the NHE1ct linker to alanines reduces its binding dynamics.*
(**A**) Overlay of the 2D [^{1}H,^{15}N] HSQC spectra of ^{15}N-labeled NHE1ct$_{\Delta92}$ in the presence (black) and absence (red) of CN (1:1 ratio). (**B**) Overlay of the 2D [^{1}H,^{15}N] HSQC spectra of ^{15}N-labeled NHE1ct$_{\Delta92RARA}$ (^{698}RAR^{700} to AAA) in the presence (black) and absence (red) of CN (1:1 ratio).

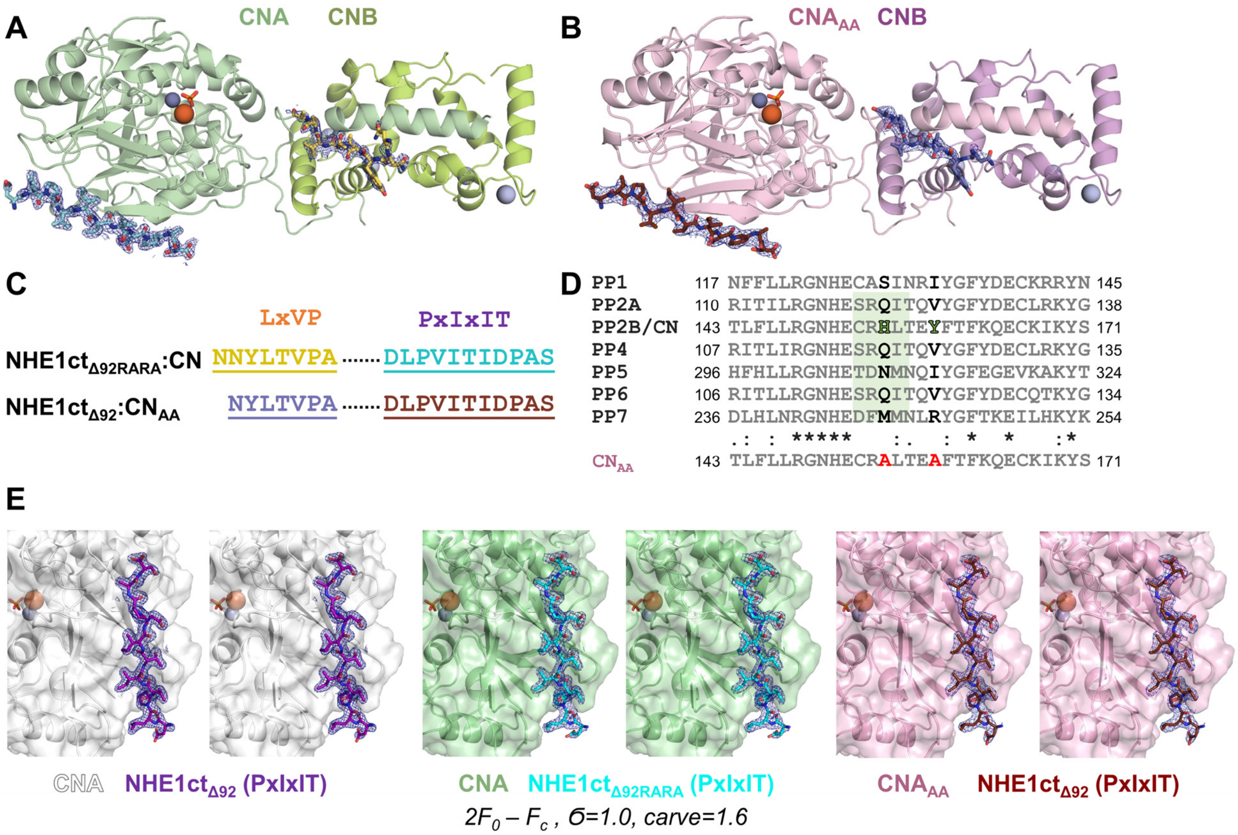

Supplementary Figure 5. *Crystal structures of NHE1ct$_{Δ92RARA}$:CN (^{698}RAR^{700} to AAA) and NHE1ct$_{Δ92}$:CN$_{AA}$ (H155A/Y159A).*

(**A**) Crystal structure of NHE1ct$_{Δ92RARA}$:CN. NHE1ct is shown as yellow (NHE1 N681-A688, LxVP motif) or cyan (NHE1 D713-S723, PxIxIT motif) sticks. CN is shown as cartoon (CNA, pale green, CNB: lime green). The $2F_o - F_c$ electron density map is contoured at 1σ (blue mesh); bound metals are shown as spheres (Fe: brown, Zn: slate, Ca: light blue). (**B**) Crystal structure of NHE1ct$_{Δ92}$:CN$_{AA}$. NHE1 is shown as slate (NHE1 N682-A688, LxVP motif) or brown (NHE1 D713-S723, PxIxIT motif) sticks. CN is shown as cartoon (CNA, light pink, CNB: light purple). (**C**) Strong electron density was observed for CN and a portion of NHE1, allowing the following NHE1 amino acids to be modeled: NHE1 N681-D688 and NHE1 D713-S723 (NHE1ct$_{Δ92RARA}$:CN); NHE1 N682-A688 and NHE1 D713-S723 (NHE1ct$_{Δ92}$:CN$_{AA}$).(**D**) Sequence alignment of human Ser/Thr phosphatases around the i+3 Pro binding pocket of CN. Residue numbers correspond to the alpha isoform of each protein. (**E**) Stereo image of NHE1ct$_{Δ92}$:CN (left panel), NHE1ct$_{Δ92RARA}$:CN (middle panel) and NHE1ct$_{Δ92}$:CN$_{AA}$ (right panel) crystal structures. $2F_o - F_c$ electron density maps are contoured at 1σ (blue mesh); bound metals are shown as spheres (Fe^{2+}: brown, Zn^{2+}: slate); phosphate is shown as sticks. CNA is shown as cartoon and a transparent surface and NHE1 (D713-S723) is shown as sticks.

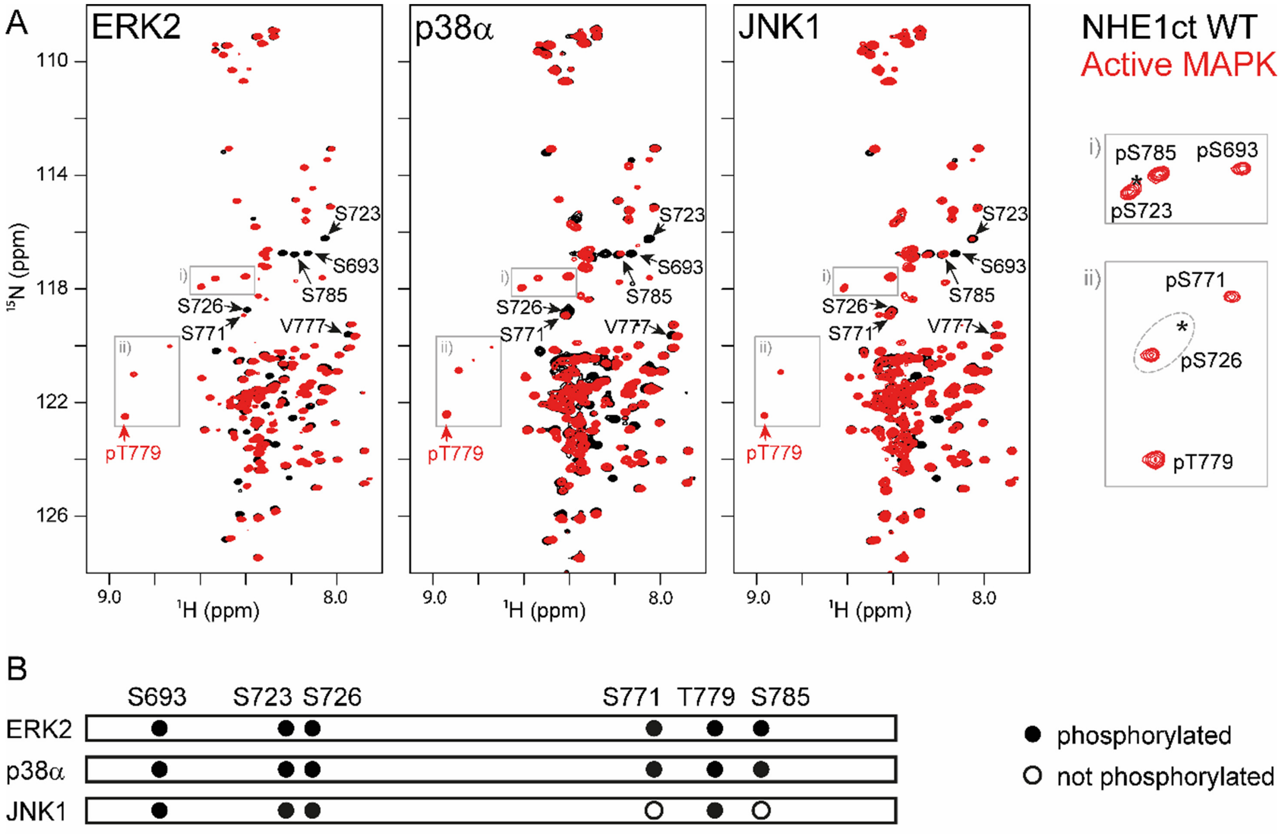

Supplementary Figure 6. *Phosphorylation of NHE1ct by various MAPKs display a similar phosphorylation pattern.*

(**A**) Overlay of extracts from the 2D [^{1}H,^{15}N] HSQC spectra of NHE1ct before (black), and after the addition of ERK2, p38α, or JNK1 (red). Addition of MAPKs resulted in new peaks for the phosphorylated state of NHE1ct and shifts of peaks due to phosphorylation of neighboring residues (e.g. V777). T779 is phosphorylated by all three kinases. Phosphorylated residues by ERK2 are highlighted on the most right panel. (**B**) Scheme summary of (**A**). Phosphorylated residues are highlighted by filled circles.

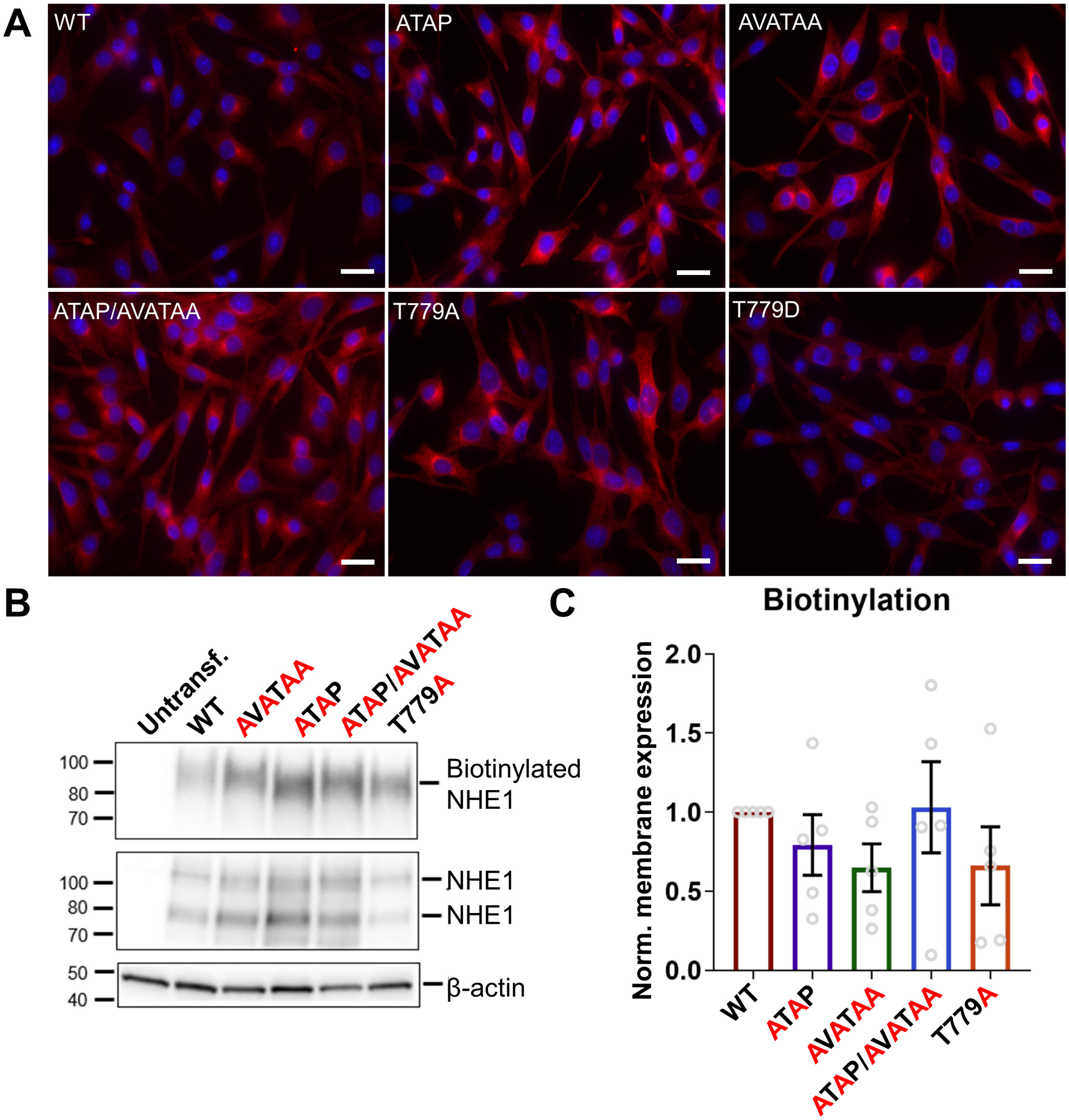

Supplementary Figure 7. *NHE1 variants localize to the plasma membrane.*
(**A**) Representative immunofluorescence (IF) images of the six variant PS120 cell lines stained for NHE1 (red) and DAPI (blue) to evaluate NHE1 expression and localization. Images were captured at 60x magnification using an Olympus Cell Vivo microscope. Scale bars represent 20 µm (n=2). (**B**) Cell surface expressison of NHE1 variants in PS120 cell lines determined by cell surface biotinylation. The lower band corresponds to immature unglycosylated NHE1 and the upper band to glycosylated NHE1. β-actin was used as loading control. (**C**) Normalized membrane expression of NHE1 variants, quantified from data as in (**B**). The biotinylated fraction was normalized to total NHE1 expression and all data normalized to WT control. No statistical differences are identified by one-way ANOVA (*n=5*). Error bars represent S.E.M. Source data are provided as a Source Data file.

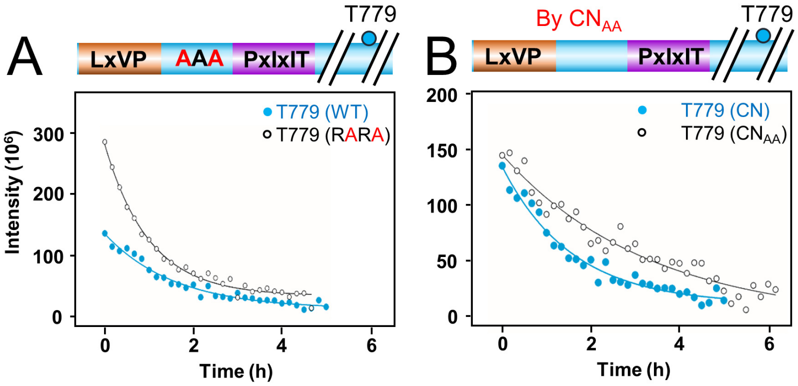

Supplementary Figure 8. *Dephosphorylation of NHE1ct measured by NMR spectroscopy.*
(**A**) Time course of T779 dephosphorylation of NHE1ct$_{WT}$ and NHE1ct$_{RARA}$ (^{698}RAR^{700} to AAA). (**B**) T779$_{NHE1}$ dephosphorylation by CN compared to CN$_{AA}$. Source data are provided as a Source Data file.

Supplementary Table 1. Data collection and refinement statistics (molecular replacement).

	NHE1ct$_{\Delta 92}$:CN[a]	NHE1ct$_{\Delta 92RARA}$:CN[a]	NHE1ct$_{\Delta 92}$:CN$_{AA}$[a]
**Data collection**			
Space group	C 2 2 2$_1$	C 2 2 2$_1$	C 2 2 2$_1$
Cell dimensions			
a, *b*, *c* (Å)	79.6, 127.1, 127.4	79.5, 126.8, 127.4	78.8, 125.5, 126.2
α, β, γ (°)	90, 90, 90	90, 90, 90	90, 90, 90
Resolution (Å)	37.97 – 1.90 (1.94– 1.90)*	16.13 - 1.90 (1.95-1.90)*	20.79 - 2.30 (2.38-2.30)*
R_{merge}	0.086 (0.923)	0.065 (0.501)	0.218 (0.773)
$I / \sigma I$	13.6 (1.9)	7.0 (1.3)	5.9 (2.3)
Completeness (%)	98.8 (96.4)	97.6 (83.0)	99.8 (100.0)
Redundancy	7.5 (7.5)	2.7 (2.0)	5.3 (5.3)
$CC_{1/2}$	0.977 (0.959)	0.997 (0.751)	0.984 (0.714)
**Refinement**			
Resolution (Å)	37.97 - 1.90 (1.97 - 1.90)	16.13 - 1.90 (1.97 - 1.90)	20.80 - 2.30 (2.38 - 2.30)
No. reflections	50470	49548	27309
R_{work} / R_{free}	0.16(0.24)/0.19(0.29)	0.17(0.26)/0.21(0.32)	0.20(0.28)/0.24(0.36)
No. atoms			
Protein	4346	4327	4292
Ligand/ion	19	39	18
Water	436	471	256
B-factors			
Protein	33.7	25.6	35.0
Ligand/ion	37.5	40.6	31.7
Water	38.8	31.2	33.2
R.m.s. deviations			
Bond lengths (Å)	0.005	0.016	0.006
Bond angles (°)	0.71	1.06	0.69

[a]Data was collected from a single crystal

*Values in parentheses are for highest-resolution shell

Supplementary Table 2. Apparent *in vitro* dephosphorylation rates of NHE1 by CN (k_{dephos} [10^{-3} h^{-1}]).

	CN				CNA*
NHE1ct variant	WT	$NHE1ct_{AVATAA}$	$NHE1ct_{ATAP}$	$NHE1ct_{ATAP/AVATAA}$	WT
*p*S693	< 1	< 1	< 1	21 ± 2	< 1
*p*S723	< 1	< 1	< 1	79 ± 4	< 1
*p*S726	< 1	< 1	< 1	67 ± 4	< 1
*p*S771	< 1	< 1	187 ± 21	ND#	107 ± 15
*p*T779	98 ± 3	11 ± 3	642 ± 28	811 ± 55	347 ± 10
*p*S785	< 1	< 1	79 ± 5	24 ± 4	37 ± 4

*Dephosphorylation of NHE1ct WT by CNA only, which lacks the LxVP binding site.
#S771 was not analyzed due to inadequate data quality.
Apparent rates of dephosphorylation and associated errors are extracted from global non-linear least square fits of disappearing peaks (dephosphorylated) in time-dependnent 2D [^1H,^{15}N] HSQC spectra to single exponentials using SigmaPlot. Source data are provided as a Source Data file.

Supplementary Table 3. T_m and Michaelis–Menten kinetics for CN and CN_{AA}.

CN variants*	Thermal Stability	Michalis-Menten kinetic constants		
	T_m (°C)	k_{cat} (s^{-1})	K_m (μM)	k_{cat}/K_m
**WT**	63.7 ± 0.9	1.7 ± 0.1	326 ± 29	5.2 ± 0.6
**CN_{AA} (H155A, Y159A)**	65.0 ± 1.0	1.7 ± 0.1	328 ± 30	5.1 ± 0.6

*CNA (M1-A391), CNB (M1-V170) (n=3). Source data are provided as a Source Data file.

Supplementary Table 4. Primers and recombinant DNA

REAGENT or RESOURCE	SOURCE
Oligonucleotides	
Primer: NHE1 LTVP-ATAP$_{(684-687)}$ Forward:<br>5’ gatcaacaactacgcgacggcgccagcccacaa 3’	Hendus-Altenburger et al., 2016[1]
Primer: NHE1 LTVP-ATAP$_{(684-687)}$ Reverse:<br>5’ cttgtgggctggcgccgtcgcgtagttgttgatc 3’	Hendus-Altenburger et al., 2016[1]
Primer: NHE1 PVITID-AVATAA$_{(715-720)}$ Forward:<br>5’ ggaggacctggctgtcgccaccgccgccccggcttcc 3’	This paper
Primer: NHE1 PVITID-AVATAA$_{(715-720)}$ Reverse:<br>5’ ggaagccggggcggcggtggcgacagccaggtcctcc 3’	This paper
Primer NHE1 PVITID-PVIVIT$_{(715-720)}$ Forward:<br>5’ ggaggacctggctgtcgccaccgccgccccggcttcc 3’	This paper
Primer NHE1 PVITID-PVIVIT$_{(715-720)}$ Reverse:<br>5’ ggaagccggggcggcggtggcgacagccaggtcctcc 3’	This paper
Primer: NHE1 T779S Forward:<br>5’ cgatgtcttctcccccgcgcccag 3’	This paper
Primer: NHE1 T779S Reverse:<br>5’ ctgggcgcgggggagaagacatcg 3’	This paper
Primer: NHE1 T779A Forward:<br>5’ cgatgtcttcgcccccgcgcccagtgac 3’	This paper

Primer: NHE1 T779A Reverse:<br>5’ gtcactgggcgcgggggcgaagacatcg 3’	This paper
Primer: NHE1 T779D Forward:<br>5’ cgatgtcttcgaccccgcgcccagtgac 3’	This paper
Primer: NHE1 T779D Reverse:<br>5’ gtcactgggcgcggggtcgaagacatcg 3’	This paper
Primer: NHE1 P782A Forward:<br>5’ ccccgcggccagtgacagccccagc 3’	This paper
Primer: NHE1 P782A Reverse:<br>5’ gctggggctgtcactggccgcgggg3’	This paper
Primer: NHE1 S785T Forward:<br>5’ gcccagtgacacccccagctcccag 3’	This paper
Primer: NHE1 S785T Reverse:<br>5’ ctgggagctgggggtgtcactgggc 3’	This paper
Primer: NHE1 SPSS-TPAP$_{(785-788)}$ Forward:<br>5’ ccagtgacacccccgccccgcagaggatacagcg 3’	This paper
Primer: NHE1 SPSS-TPAP$_{(785-788)}$ Reverse:<br>5’ cgctgtatcctctgcggggcgggggtgtcactgg 3’	This paper
Cloning primer NHE1 680 Start Forward:<br>5’ attcgccatatgaagatcaacaactacctgacgg 3’	This paper
Cloning primer NHE1 723 End Reverse:<br>5’ gctaaccctcgagtcaggaagccgggtcgatggtg 3’	This paper
Cloning primer NHE1 815 End Reverse:<br>5’ gctaaccctcgagtcactgccccttggggaag 3’	This paper
Primer: NHE1 RARA (R678A, R700A) Forward:<br>5’ ggactcacccaccatgtctgcggccgccatcggctcagaccc 3’	This paper
Primer: NHE1 RARA (R678A, R700A) End Reverse:<br>5’ gggtctgagccgatggcggccgcagacatggtgggtgagtcc 3’	This paper
Cloning primer: Calcineurin A 27 Forward:<br>5’ ggcggatcccaccggctt 3’	This paper
Cloning primer: Calcineurin A 348 Reverse:<br>5’ ttcatggatgacgattaactcgaggcc 3’	This paper
Primer: add DD C-terminal to Calcineurin A 27-348 Forward:<br>5’ tactggcttccaaatttcatggatgacgattaactcgagcacc 3’	This paper
Primer: add DD C-terminal to Calcineurin A 27-348 Reverse:<br>5’ ggtgctcgagttaatcgtcatccatgaaatttggaagccagta3’	This paper
Primer: Calcineurin H155A, Y159A Forward:<br>5’<br>cttcgtggaaatcatgaatgtagagcactaacagaggctttcacatttaaaca agaatgt 3’	This paper
Primer: Calcineurin H155A, Y159A Reverse:<br>5’<br>acattcttgtttaaatgtgaaagcctctgttagtgctctacattcatgatttccacg aag 3’	This paper
Primer: CNB 15 Forward:<br>5’ tttaatttcatccgcatcaaacatatgtatatctcctcgagtcagg 3’	This paper
Primer: CNB 15 Reverse:<br>5’ cctgactcgaggagatatacatatgtttgatgcggatgaaattaaa 3’	This paper
Primer: CNB 16 Forward:<br>5’ ctgactcgaggagatatacatatggatgcggatgaaatt 3’	This paper
Primer: CNB 16 Reverse:<br>5’ aatttcatccgcatccatatgtatatctcctcgagtcag 3’	This paper
Recombinant DNA	

pET11.a NHE1cdt WT<br>(human NHE1 680-815)	Hendus-Altenburger et al., 2016[1]
pET11.a NHE1cdt ATAP<br>(human NHE1 680-815)	Hendus-Altenburger et al., 2016[1]
pET11.a NHE1cdt AVATAA<br>(human NHE1 680-815)	This paper
pET11.a NHE1cdt ATAP+AVATAA<br>(human NHE1 680-815)	This paper
pET11.a NHE1cdt T779S<br>(human NHE1 680-815)	This paper
pET11.a NHE1cdt P782A<br>(human NHE1 680-815)	This paper
pET11.a NHE1cdt S785T<br>(human NHE1 680-815)	This paper
pET11.a NHE1cdt S785-TPAP<br>(human NHE1 680-815)	This paper
pcDNA3.1 Full length NHE1 WT<br>(human NHE1 1-815)	Pedersen et al., 2007[2]
pcDNA3.1 Full length NHE1 ATAP<br>(human NHE1 1-815)	Hendus-Altenburger et al., 2016[1]
pcDNA3.1 Full length NHE1 AVATAA<br>(human NHE1 1-815)	This paper
pcDNA3.1 Full length NHE1 ATAP+AVATAA<br>(human NHE1 1-815)	This paper
pcDNA3.1 Full length NHE1 T779A<br>(human NHE1 1-815)	This paper
pcDNA3.1 Full length NHE1 T779D<br>(human NHE1 1-815)	This paper
pET-M30-MBP NHE1ct WT<br>(human NHE1 680-815)	This paper
pET-M30-MBP NHE1ct RARA<br>(human NHE1 680-815, R678A, R700A)	This paper
pET-M30-MBP NHE1ct Δ92<br>(human NHE1 680-723)	This paper
pET-M30-MBP NHE1ct Δ92 RARA<br>(human NHE1 680-723, R678A, R700A)	This paper
Calcineurin A (alpha isoform, subunit A: 27-348DD)	This paper
Calcineurin A/B (alpha isoform, subunit A: 1-370,<br>subunit B: 1-170)	Grigoriu, Bond et al. 2013[3]
Calcineurin A/B (alpha isoform, subunit A: 1-391,<br>subunit B: 1-170)	Grigoriu, Bond et al. 2013[3]
Calcineurin A/B (alpha isoform, subunit A: 1-391,<br>H155A, Y159A, subunit B: 1-170)	This paper
Calcineurin A/B (alpha isoform, subunit A: 1-370,<br>subunit B: 16-170)	This paper
Calcineurin A/B (alpha isoform, subunit A: 1-370,<br>H155A, Y159A, subunit B: 16-170)	This paper

Supplementary References

1. Hendus-Altenburger, R. *et al.* The human Na(+)/H(+) exchanger 1 is a membrane scaffold protein for extracellular signal-regulated kinase 2. *BMC Biol.* **14**, 31 (2016).

2. Pedersen, S. F., King, S. A., Nygaard, E. B., Rigor, R. R. & Cala, P. M. NHE1 inhibition by amiloride- and benzoylguanidine-type compounds. Inhibitor binding loci deduced from chimeras of NHE1 homologues with endogenous differences in inhibitor sensitivity. *J. Biol. Chem.* **282**, 19716–19727 (2007).

3. Grigoriu, S. *et al.* The molecular mechanism of substrate engagement and immunosuppressant inhibition of calcineurin. *PLoS Biol.* **11**, e1001492 (2013).
