# Supplementary material for: Molecular basis for the binding and selective dephosphorylation of Na+/H+ exchanger 1 by calcineurin
Source: Nat Commun. 2019 Aug 2;10:3489. doi: 10.1038/s41467-019-11391-7 (PMC6677818; doi:10.1038/s41467-019-11391-7)
Supplement: Supplementary file 3 — Reporting Summary [file 41467_2019_11391_MOESM3_ESM.pdf]

nature research

Corresponding author(s): Rebecca Page, Birthe B. Kragelund, Stine F. Pedersen, Wolfgang Peti

Last updated by author(s): Jul 3, 2019

Reporting Summary

Nature Research wishes to improve the reproducibility of the work that we publish. This form provides structure for consistency and transparency in reporting. For further information on Nature Research policies, see Authors & Referees and the Editorial Policy Checklist.

Statistics

For all statistical analyses, confirm that the following items are present in the figure legend, table legend, main text, or Methods section.

n/a	Confirmed	
☐	☒	The exact sample size (*n*) for each experimental group/condition, given as a discrete number and unit of measurement
☐	☒	A statement on whether measurements were taken from distinct samples or whether the same sample was measured repeatedly
☐	☒	The statistical test(s) used AND whether they are one- or two-sided<br>*Only common tests should be described solely by name; describe more complex techniques in the Methods section.*
☒	☐	A description of all covariates tested
☐	☒	A description of any assumptions or corrections, such as tests of normality and adjustment for multiple comparisons
☐	☒	A full description of the statistical parameters including central tendency (e.g. means) or other basic estimates (e.g. regression coefficient) AND variation (e.g. standard deviation) or associated estimates of uncertainty (e.g. confidence intervals)
☐	☒	For null hypothesis testing, the test statistic (e.g. F, t, r) with confidence intervals, effect sizes, degrees of freedom and P value noted<br>*Give P values as exact values whenever suitable.*
☒	☐	For Bayesian analysis, information on the choice of priors and Markov chain Monte Carlo settings
☒	☐	For hierarchical and complex designs, identification of the appropriate level for tests and full reporting of outcomes
☒	☐	Estimates of effect sizes (e.g. Cohen's d, Pearson's r), indicating how they were calculated

Our web collection on statistics for biologists contains articles on many of the points above.

Software and code

Policy information about availability of computer code

Data collection	CellSense, FluoStar Optima software, Topspin, VnmrJ
Data analysis	ImageJ, GraphPad Prism, NMRPipe, CCPN Analysis, SigmaPlot 13.0, excel

For manuscripts utilizing custom algorithms or software that are central to the research but not yet described in published literature, software must be made available to editors/reviewers. We strongly encourage code deposition in a community repository (e.g. GitHub). See the Nature Research guidelines for submitting code & software for further information.

Data

Policy information about availability of data

All manuscripts must include a data availability statement. This statement should provide the following information, where applicable:

- Accession codes, unique identifiers, or web links for publicly available datasets
- A list of figures that have associated raw data
- A description of any restrictions on data availability

The source data underlying Figs 2c-2j, 3b, 4b-g, 5b-h and Supplementary Figs 3a-c, 7, 8a-8b and Supplementary Table 2 and 3 are provided as a Source Data file.

Field-specific reporting

Please select the one below that is the best fit for your research. If you are not sure, read the appropriate sections before making your selection.

☒ Life sciences ☐ Behavioural & social sciences ☐ Ecological, evolutionary & environmental sciences

For a reference copy of the document with all sections, see nature.com/documents/nr-reporting-summary-flat.pdf

Life sciences study design

All studies must disclose on these points even when the disclosure is negative.

Sample size	Sample size was chosen based on previous experience with effect size
Data exclusions	No data were excluded
Replication	All data are based on multiple independent replications
Randomization	N/A
Blinding	N/A

Reporting for specific materials, systems and methods

We require information from authors about some types of materials, experimental systems and methods used in many studies. Here, indicate whether each material, system or method listed is relevant to your study. If you are not sure if a list item applies to your research, read the appropriate section before selecting a response.

Materials & experimental systems

n/a	Involved in the study
☐	☒ Antibodies
☐	☒ Eukaryotic cell lines
☒	☐ Palaeontology
☒	☐ Animals and other organisms
☒	☐ Human research participants
☒	☐ Clinical data

Methods

n/a	Involved in the study
☒	☐ ChIP-seq
☒	☐ Flow cytometry
☒	☐ MRI-based neuroimaging

Antibodies

Antibodies used

Primary antibody: mouse monoclonal anti-NHE1, NHE-54 Santa Cruz Cat#sc-136239
Primary antibody: polyclonal anti-CNA Cell Signaling Cat#5530, Cat#5532
Primary antibody: rabbit polyclonal anti-NHE1 Mark Musch, University of Chicago XB-17
Primary antibody: rabbit polyclonal anti-CNB Biomol Cat#B2336
Primary antibody: mouse monoclonal anti-beta-actin Sigma Cat#AB_476744
Primary antibody: mouse monoclonal anti-NHE1 clone 4e9, MAB3140, MerckMilipore
Primary antibody: mouse monoclonal anti-CNA, Sigma-Aldrich, Cat#1956
Primary antibody: mouse monoclonal anti-p150Glued, BD Biosciences, Cat#BD610473
IgG isotype controls: Cell Signaling Technology, Cat#CS2729 and Cat#CS9706 (pH3)
Horse radish peroxidase (HRP)-conjugated secondary antibody Dako Cat#P0447 (mouse) and Cat#P0448 (rabbit)
AlexaFluor568-conjugated secondary antibody ThermoFisher Cat#A10037 (mouse) and Cat#A10042 (rabbit)

Validation

All are commercial and validated by the company, except Xb-17, which is validated in NHE1 KD and KO cells in our own laboratory.

Eukaryotic cell lines

Policy information about cell lines

Cell line source(s)

MCF-7 cells Dr. Lone Ronnov-Jessen, University of Copenhagen, Denmark;
PS120 cells, Prof. L. Counillon, University of Nice, France

Authentication

MCF7 cells are regularly validated by immunofluorescence and immunoblotting analysis for known markers (e.g HER2, ERK, Akt, and characteristic lack of caspase-3.
PS120 cells are routinely tested by immunofluorescence and immunoblotting analysis for continued absence of endogenous NHE1.

Mycoplasma contamination

All cell lines are routinely tested for mycoplasma every 3 months and found negative

Commonly misidentified lines
(See ICLAC register)

Name any commonly misidentified cell lines used in the study and provide a rationale for their use.
